# Changes in Black Truffle (*Tuber melanosporum*) Aroma during Storage under Different Conditions

**DOI:** 10.3390/jof10050354

**Published:** 2024-05-15

**Authors:** Ruben Epping, Jan Lisec, Matthias Koch

**Affiliations:** Department of Analytical Chemistry and Reference Materials, Bundesanstalt für Materialforschung und-Prüfung (BAM), 12489 Berlin, Germany; ruben.epping01@googlemail.com (R.E.); jan.lisec@bam.de (J.L.)

**Keywords:** truffle aroma, volatile organic compounds, gas chromatography, storage conditions, analysis of variance

## Abstract

The enticing aroma of truffles is a key factor for their culinary value. Although all truffle species tend to be pricy, the most intensely aromatic species are the most sought after. Research into the aroma of truffles encompasses various disciplines including chemistry, biology, and sensory science. This study focusses on the chemical composition of the aroma of black truffles (*Tuber melanosporum*) and the changes occurring under different storage conditions. For this, truffle samples were stored under different treatments, at different temperatures, and measured over a total storage time of 12 days. Measurements of the truffle aroma profiles were taken with SPME/GC–MS at regular intervals. To handle the ample data collected, a systematic approach utilizing multivariate data analysis techniques was taken. This approach led to a vast amount of data which we made publicly available for future exploration. Results reveal the complexity of aroma changes, with 695 compounds identified, highlighting the need for a comprehensive understanding. Principal component analyses offer initial insights into truffle composition, while individual compounds may serve as markers for age (formic acid, 1-methylpropyl ester), freshness (2-Methyl-1-propanal; 1-(methylthio)-propane), freezing (tetrahydrofuran), salt treatment (1-chloropentane), or heat exposure (4-hydroxy-3-methyl-2-butanone). This research suggests that heat treatment or salt contact significantly affects truffle aroma, while freezing and cutting have less pronounced effects in comparison. The enrichment of compounds showing significant changes during storage was investigated with a metabolomic pathway analysis. The involvement of some of the enriched compounds on the pyruvate/glycolysis and sulfur pathways was shown.

## 1. Introduction

There are several different truffle species that are consumed as food. Some are moderately expensive; some are among the most expensive foods that exist. Apart from their seasonality and rarity, the respective value is mainly attributed to their aroma [1]. The aroma is affected by species but also by the geographic origin, ripeness, climate, genetics, and soil composition [2,3,4].

There are about 30 species of truffles that are being traded commercially. The two most expensive and sought-after truffle species are the black Perigord truffle (*Tuber melanosporum*) and the white Alba truffle (*Tuber magnatum*). Most commercially relevant are *Tuber melanosporum* because they can be cultivated in truffle orchards. By inoculating the roots of young suitable trees like oak and hazelnut with truffle spores, the fungus will grow in symbiosis with the trees [5,6,7]. The smell of ripe truffles is also what attracts fungivore animals (and truffle dogs) to them [8,9]. Truffles can be found predominantly in southern Europe in countries like Italy, France, and Spain. In recent years, however, orchards have also been established in Australia, Argentina, the USA, and other places [10,11,12,13]. The life cycle of truffles is comprised of the succession of three different phases. In the first phase, the mycelia grow. Then, the ectomycorrhiza is formed. Finally, the (edible) fruiting body develops [2].

The aroma of truffle is comprised of dozens to hundreds of volatile organic compounds (VOCs). Among them are aldehydes, alcohols, ketones, organic acids, esters, terpenes, or organic sulfur compounds [14,15]. While many of these compounds, like ethanol, acetaldehyde, and acetone, can also be found in various natural aromas, others are more unique to truffles in general or for single truffle species. Often the smell of truffles is described as earthy, damp-forest-like, cooked potato leather, or sulfurous. Key odorants that were found in multiple studies are, for example, 3-methylbutanal, 2-methylbutanal, 2-methylbutan-1-ol, 1-octen-3-ol, 2-methyl-1-propanol, or anisole. It was found in previous research that organic sulfur compounds play an especially import role in the truffle aroma and may be key to the attraction and ripeness of them. Dimethyl sulfide and dimethyl disulfide were present in different truffle species, while 2,4-dithiapentane seems to be specific for *Tuber magnatum* and thiophene derivatives to *Tuber borchii* [16,17,18,19].

While these are examples of findings in multiple prior works, it is important to note that the aroma of truffles can vary greatly, not only by species but also due to soil conditions, time of harvest, microorganisms present, weather, and many other factors not yet fully understood. Additionally, the published aroma compositions are obtained utilizing different analytical techniques. While most researchers seem to use some form of gas chromatography–mass spectrometry (GC–MS) coupling [20,21,22,23,24,25], often in combination with solid-phase microextraction (SPME) or headspace sampling (HS), others also use direct-injection mass spectrometry (DIMS) [26,27,28] or other techniques [29,30,31] to obtain their results. Therefore, the aroma compositions published for truffles can vary greatly.

As food, truffles have a limited shelf life of normally only 7–10 days. This is mainly attributed to their high water content and biological activity from bacteria and mold growth [32]. In this timespan, they typically lose their enticing aroma. This is due to oxidation, enzymatic reactions, or simply degassing during storage. To combat this loss of aroma, truffles are often processed into truffle-flavored forms like truffle oils, spreads, cheese, or preserved meat products [33,34]. The preservation of their culinary attributes without these processes seems to be especially difficult.

There are some studies that investigate the possibility of preserving whole truffles through means of freezing, freeze-drying, encapsulation, canning, drying, refrigeration, pasteurization, inert atmosphere, or others. Freeze-drying seems to have been of special interest and showed to maintain some of the truffle’s aroma after rehydration. There are also studies that investigated the changes of truffle aroma during storage or in general under various conditions. Important examples of these works are:

Siskovic et al. found that freeze-drying significantly alters the VOC profile of truffle but were still able to distinguish between different species using multivariate discriminant analysis [35]. Although, the organoleptic qualities were still affected negatively by freeze-drying. For their study, they conducted HS-SPME/GC–MS measurements. A microbiological comparison of *Tuber melanosporum* stored at 4 °C whole, sliced, or freeze-dried was carried out by Phong et al. via plate counts of different microbes. They found that the total microbial count was generally lower for the freeze-dried truffles [36]. Campo et al. evaluated the preservation methods of freeze-drying, hot-air drying, freezing, and canning in a study combining the results from SPME/GC–MS and an olfactometric panel. They found that only the freeze-drying and hot-air drying processes could retain key aroma compounds [37]. Freezing truffles preserves truffles on a biological level but has been shown to negatively affect the texture and perceived aroma.

Longo et al. traced the antioxidant power of truffles stored under reduced and modified atmosphere. The reduced atmosphere was found to be better suited for storage. Also, markers for freshness (adducts of glutathione and adenine) and degradation (acetyl-carnitine adduct with cysteinyl-glycine) could be identified [38]. For their study, they used HPLC coupled with a CoulArray detector. Similarly, Savini et al. stored black truffles under various atmospheric conditions. They were able to prolong shelf lives up to two weeks and could correlate antioxidant activity with three volatile aldehydes using a mix of SPME–GC/MS and HPLC–MS/MS measurements [39].

The influence of bacteria on the aroma deterioration of *tuber aestivum* was investigated by Vahdatzadeh et al. The gradual replacement of bacteria characteristic for fresh truffles with food spoilage bacteria was observed with SPME–GC/MS measurements. Furthermore, they could also identify volatile markers for freshness and spoilage (i.e., dimethyl sulfide (DMS), butan-2-one, 2-methylbutan-1-ol, and 2-phenylethan-1-ol) [40]. In another study, Savini et al. found that hypobaric packaging at 30 kPa was able to prolong the shelf life of truffles up to 14 days considering epiphytic microbial population, firmness, weight loss, CO_2_ formation, and sensory properties [41]. For their study, they combined SPME–GC/MS with sensory and firmness measurements.

*Tuber melanosporum* samples from Australia were investigated by Choo et al. They observed significant changes in 11 key volatiles (carbon dioxide, acetaldehyde, 2-butanone, 3-methyl-1-butanal, toluene, 2-butenal, formic acid 2- methyl butyl ester, 3-methyl-1-butanol, 6-methyl-2-heptanol, 3-octanol, and dimethyl sulfoxide) in GC–MS measurements mostly in the first 7 days of storage in the fridge. Some similarities and some differences compared to the aroma of European truffles were noted [42]. *Tuber magnatum* changes during storage were studied by Niimi et al. with GC–MS and PCR-high-throughput sequencing They too found numerous changes in the aroma profile, most notably a steep reduction in the key VOC 2,4-dithiapentane [43].

Phong et al. investigated the extraction of natural truffle aromas via supercritical carbon dioxide extraction as a way to overcome the short shelf life of truffles [44]. It proved to be a viable method, although the aroma was altered to some degree.

Any form of heat treatment seems to have a strong effect on truffle aroma and organoleptic evaluations [45]. Thus, refrigeration remains the most common preservation method, even though it extends the shelf life by only a few days.

The changes in the aroma of truffles are not yet fully understood. As shown above, previous studies came to different, sometimes uncorrelated, results that are not always consistent. Because of this, we aim to expand the knowledge in this field with this work. We are interested in the changes of the aroma profile of truffles during storage, since it may be of value to develop better conservation methods and constitute an insight into the processes going on in truffles after harvest.

To do this, we focused on *Tuber melanosporum* for this investigation. To generate a large amount of data, we stored truffles under different conditions, at different temperatures, and took measurements over a total storage time of 12 days. The goal was to integrate several factors simultaneously with this experimental layout. We intended to generate dense time course data of metabolic traits in a non-targeted fashion and hypothesize that these data can be used to model the process of deterioration and, in this way, could lead to better conservation strategies.

Measurements of the truffle aroma profiles were taken with SPME/GC–MS at regular intervals. To handle the ample data collected, we took a systematic approach utilizing multivariate data analysis techniques.

## 2. Materials and Methods

### 2.1. Fruiting Bodies

Samples of *Tuber melanosporum* were purchased online (Ross Ltd., Berlin, Germany). Upon receipt, the truffle samples were immediately visually and sensorially checked for authenticity of the variety and freshness.

### 2.2. Sample Preparation

For the storage experiment, samples were prepared on the day of arrival. The shipment contained 150 g of *Tuber melanosporum* in 5 roughly equally sized specimens. For each sample, 0.5 g of *Tuber melanosporum* was weighed in and stored inside a hermetically sealed 10 mL headspace vial with a septum. To ensure homogenization, all specimens were cut and randomized first. Although cutting the truffle samples inevitably leads to a faster loss of aroma compared to whole truffles, it was necessary to ensure equal-weighted portions and to fit samples into headspace vials.

Samples were stored for 12 days total and with measurements taken every 2 days including day 0. Therefore, 7 rounds of measurements were taken on days 0, 2, 4, 6, 8, 10, and 12.

Samples were stored at 3 different temperatures. The room temperature (RT) was 21 ± 1.5 °C, refrigerated temperature (KS) was 8 ± 0.5 °C, and freezer temperature was −30 ± 0.2 °C.

Furthermore, samples were stored after 4 different processing types. Sliced samples (S) were cut into 0.5 mm thin slices with a truffle slicer. Pieces of truffle (B) were broken off from truffles and cut to the exact weight. As a method for enzymatic inhibition, another part of the sliced truffle samples was sealed with the addition of 1 g of NaCl. Lastly, a portion of the samples were blanched for 2 min in 90 °C distilled water. Afterward, the blanched samples were dried with paper towels, sliced, weighed, and sealed.

For each sample, a repeat measurement was prepared. In total, for the 3 temperatures × 4 processing types × 7 measurement time points × 2 repetitions, 168 samples were prepared and stored.

Due to the long duration for each measurement cycle (60 min) and number of samples, on each measuring day, the samples were measured in the same order and taken out of storage consecutively as close to their measuring time as possible. All measured samples are listed in Table A1 in order of measurement.

### 2.3. Gas Chromatography–Mass Spectrometry (GC–MS)

For the GC–MS measurements, the autosampler Gerstel MPS—Multi Purpose Sampler (Gerstel, Mülheim an der Ruhr, Germany) was used. All samples were transferred to the agitator at 40 °C for 15 min before injection.

For the SPME measurement, the SPME fiber 50/30 microm PVB/CAR/PDMS, Stabflex by Supelco (Sigma-Aldrich Chemie GmbH, Taufkirchen, Germany) was used. Before and after each splitless injection, the fiber was baked out at 200 °C for 15 min. The loading of the fiber was carried out for 5 min at 40 °C. Desorption in the GC–MS apparatus took place for 45 s at a temperature of 200 °C.

The Agilent 7890B gas chromatograph (Agilent Technologies, Waldbronn, Germany) was equipped with a VF-624 ms column (60 m × 0.32 mm ID, 1.8 µm film thickness, Agilent, Waldbronn, Germany)). The column is specially designed for volatile and semi-volatile compounds. Helium 5.0 was used as the carrier gas (2.22 mL/min, constant flow). After the injection, the temperature of 40 °C was kept in the column oven for 15 min. This was followed by a temperature increase of 5 °C/min up to a temperature of 180° C. Finally, the temperature was increased by 25 °C/min up to a temperature of 220 °C and held for 15 min, resulting in a total run time of 59.6 min.

The eluting substances were detected with an Agilent Technologies 5977A mass spectrometer using EI ionization at 70 eV and nominal mass precision. In scan mode, 30 m/z was set as the start mass and 300 m/z as the end mass. The analysis frequency was 6 scans/s at a speed of 1.562 u/s. The gain factor was set to 2.

### 2.4. Data Analysis

For data collection, the software Chemstation version C.01.05 (Agilent) was used. All files were recorded using the .D format.

Further data processing was conducted utilizing the software tool MS-Dial v. 4.9.221218 (RIKEN Center for Sustainable Resource Science, Yokohama, Kanagawa, Japan), which is free to download (http://prime.psc.riken.jp/compms/msdial/main.html, accessed on 1 March 2024).

GC–MS data were imported into MS-DIAL with a mass range of 30–300 Da and Retention times of 0–65 min. Spectral deconvolution, alignment, and peak identification were performed using the following parameters: Peak detection parameters: minimum peak height: 1000 amplitude; smoothing method: linear-weighted moving average, with 3 scans smoothing level; deconvolution parameters: sigma window value: 0.5, EI spectra cutoff: 10 amplitude; peak identification settings: utilizing Retention Indices (RI), Index type Alkanes, RI tolerance: 100, Retention time tolerance: 2.5 min, m/z tolerance: 0.5 Da, EI similarity cutoff: 85%, identification cutoff: 70%, use retention information for scoring but not for filtering. For this purpose, previously recorded Kovats retention indices were measured by interpolating the measured retention times between those of adjacent n-alkanes. The measured retention indices (RI) were compared with the literature-known RIs derived from the NIST v17 library. The peak identifications were then verified by hand. The retention order and the plausibility of the occurrence of the substance in the sample type were considered as soft decision criteria in case of multiple potential or in case of dubious annotations.

Afterwards, the alignment results matrix was exported as an .msp file. Further statistical computing and graphics were carried out with the software R v. 4.3.2 (R Core Team) available online for free (https://www.r-project.org/, accessed on 1 March 2024) using the MetabolomicsBasics package v.1.4.5 [46].

The data analytics combining the results with biological processes were performed with MetaboAnalyst 6.0, available online for free (https://dev.metaboanalyst.ca/home.xhtml, accessed on 1 March 2024). For this, a cleaned-up data matrix was used as input. The two modules Enrichment Analysis and Pathway Analysis were utilized for sets of samples filtered by treatment process and storage temperature.

The Quantitative Enrichment Analysis was conducted using the storage times as a continuous group label. The samples were normalized by median and log10-transformed. The enrichment test was performed using Super-class and Main-class chemical structures libraries containing at least 2 entries.

The Pathway analysis was performed with the same parameters, except the pathway library Escherichia coli K-12 MG1655 was selected.

## 3. Results

### 3.1. Storage of Samples

The presented work aims to investigate changes in the truffle aroma during storage. For this, we decided to focus on *Tuber melanosporum* as it is the most commercially relevant truffle species and known for its intense and complex aroma.

To allow modelling of the deterioration process, we decided to use 21 °C, 8 °C, and −30 °C as temperature levels. We hypothesize that the comparison of time course metabolic profiles at preserving (−30 °C) and unfavorable (21 °C) conditions will allow to identify the metabolic processes causal for aroma degradation. As storage conditions, it was decided to firstly investigate the transition of volatile organic compounds (VOCs) into the gas phase. For this, the comparison between pre-sliced and chunks of truffles was chosen. The effect of the enhanced surface area of the sliced samples on the facilitation of VOC migration to the gas phase, and hence faster aroma degradation phase, was to be investigated. To study the effect of enzymatic inhibition and dry-out of samples, the storage with added NaCl was performed. Lastly, to gain insight into the effect of heat treatment, blanched samples were added to the sample pool.

The storage experiment was carried out over 12 days since this is the usual maximum shelf life of truffles. Measurements were taken only every other day as a full set of measurements took longer than 24 h, therefore measurements could not be carried out every day. More details of the sample preparation and (HS)SPME/GC–MS measurements during storage times are described in Section 2.2.

### 3.2. Data Treatment

**Figure 1:**
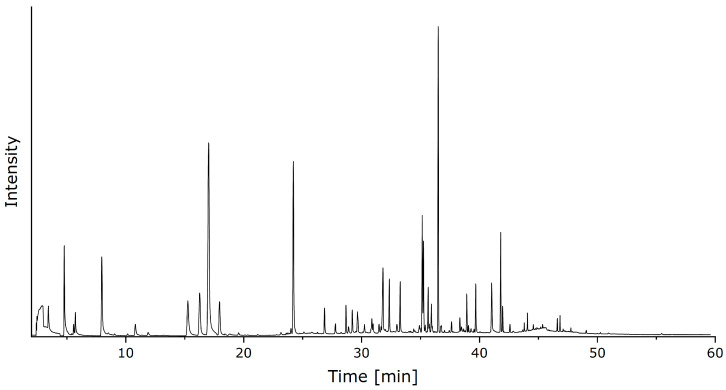
Typical (HS)SPME/GC–MS total ion chromatogram of *Tuber melanosporum*.

A typical (HS)SPME/GC–MS chromatogram of *Tuber melanosporum* is shown in Figure 1. Since only relative changes in concentrations of VOC compounds were of interest in this study, a calibration was omitted and would not have been feasible with respect to the number of compounds annotated.

**Figure A1:**
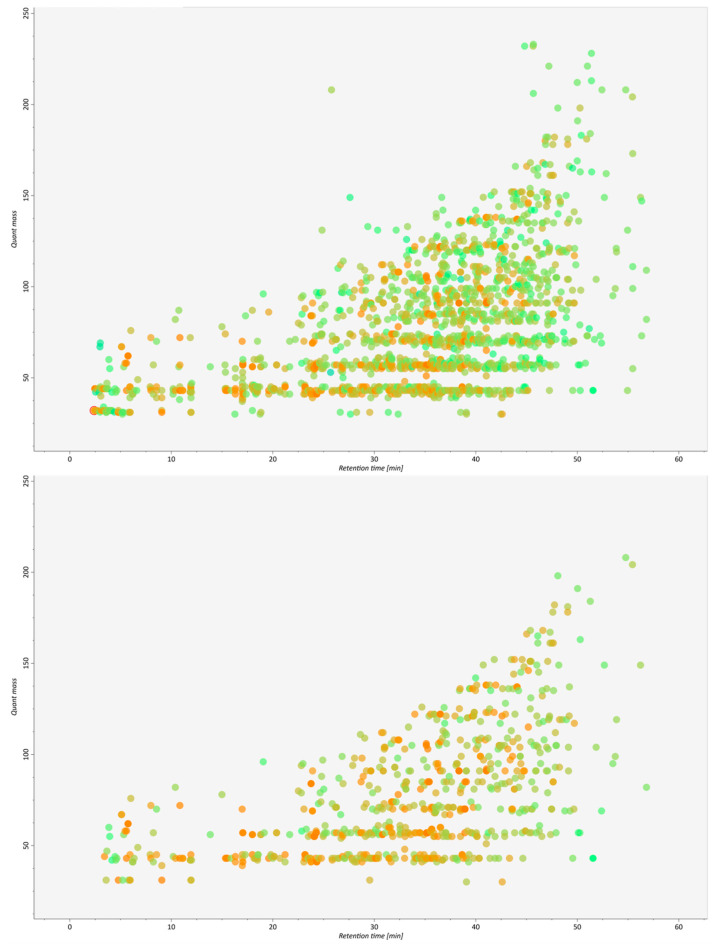
Alignment results of the MS-DIAL deconvolution process. All peaks (**top**) and identified peaks (**bottom**) in comparison with NIST library.

All chromatographic and mass spectrometric data were loaded into MS-DIAL for further processing. The tool was developed for untargeted metabolomics for multiple instruments and vendors [47]. It allows for spectral deconvolution and peak identifications [48]. The deconvolution combines individual peaks into compound spectra, which is sometimes difficult for co-eluting compounds. A beneficial side effect is the removal of noise ions. Details of the program’s settings used can be found in Section 2.4. After deconvolution, 1626 spectra could be identified (see Figure A1). Utilizing spectral and retention index comparison with NIST library, 695 compounds could be annotated.

**Figure 2:**
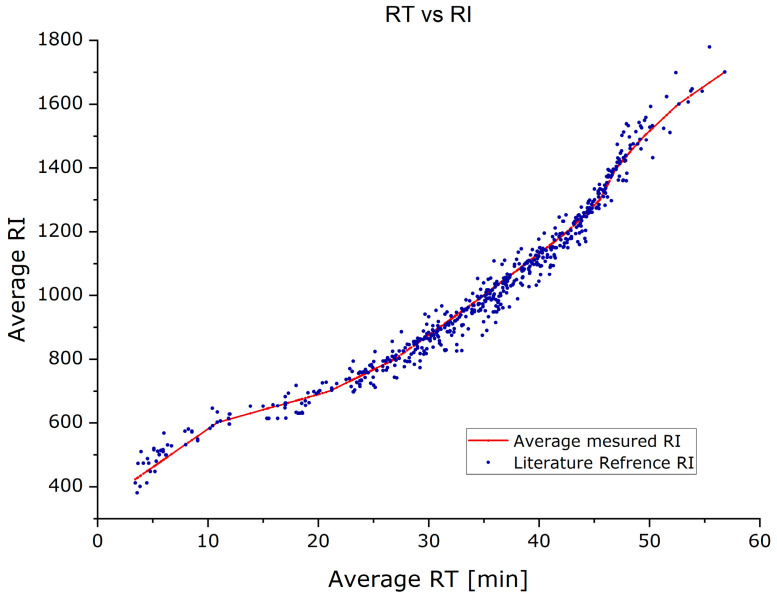
Retention time vs. retention index plot for measured RI and reference from the literature.

To check for the quality of matches, the retention time (RT) was plotted versus the retention index (RI) for RIs that were measured and RIs from the literature. The plot is shown in Figure 2. The reason for the measured plot (red) not being linear is the programmed temperature protocol of the GC run. Overall, comparing measured to the literature values, however, they are in good agreement. The matrix with peak integrations values for all identified peaks for all measured samples was then exported for further processing and is provided online (https://doi.org/10.5281/zenodo.10886445, accessed on 1 March 2024).

### 3.3. Analysis of Variance

#### 3.3.1. Methodological Verification

**Figure 3:**
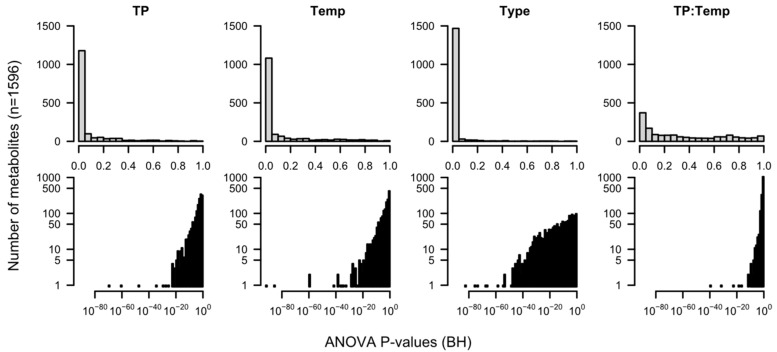
*p*-value histograms for dependence on time point (TP), temperature, processing type (condition), and both TP and temperature. (**Top**): linear, (**Bottom**): logarithmic scale. *p*-values are multiple-testing-corrected using the method of Benjamini–Hochberg (BH).

For all deconvoluted spectra (metabolites), ANOVA statistics were performed from which *p*-values were calculated [49]. The experimental factors included in the ANOVA model are the sampling time point (TP), the storage temperature level (Temp), and the sample processing type (Type). Additionally, we included an interaction term (TP:Temp) that tests for significant differences in time, dependent on the temperature level. The obtained probability (*p*)-values describe how likely the null hypothesis, meaning no relationship between the variables of interest or no differences between groups, is. The lower the *p*-value, the lower the probability of the null hypothesis being true. All *p*-values are summarized in a histogram in Figure 3 after multiple-testing correction. A value of <0.05 is usually considered sufficient to reject the null hypothesis [50].

Due to the large sample number, which results in a high statistical power of the test, we obtained significant *p*-values for most metabolites. It means that the concentration of the compounds is indeed statistically dependent of the variables TP, Temp, Type, and the interaction of TP and Temp, with Type being the most influential factor (largest number of *p*-values < 0.05 and larger number of *p*-values < 10^−40^).

**Figure 4:**
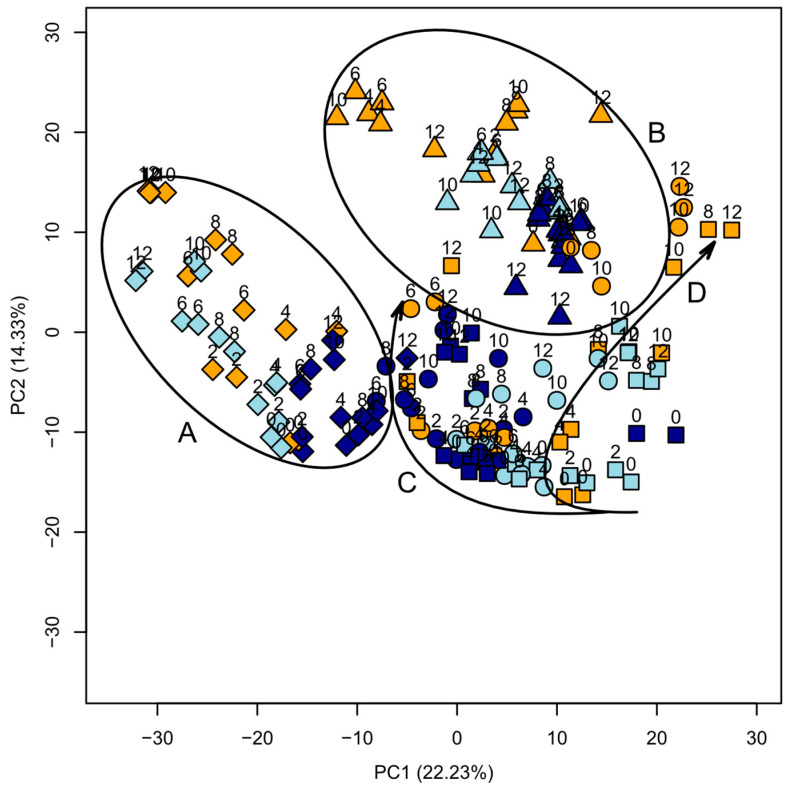
Principal component analysis of stored truffle samples. Storage time (d) is indicated by numbers. Storage temperatures of room temperature, fridge, and freezer are indicated by colors. Processing types sliced (●), piece (■), with NaCl (◆), and blanched (▲) are indicated by symbols.

The obtained data in this work correlate different samples (temperature, processing type, storage time) in many variables (metabolites). With this many dimensions, the number of possible combinations of samples increases exponentially. To reduce the dimensionality of the dataset while still preserving the relationship between samples, a principal component analysis (PCA) was performed [51]. With this, the interrelations between the samples can be observed in a simple two-dimensional score plot shown in Figure 4.

Figure 4 shows several observations that can be considered first indicators for the differences in the truffle aromas during the storage experiment. All samples treated with NaCl are clustered together and are clearly separated from other samples (marked with A). Within these samples, frozen samples are closely grouped together, indicating only small differences among them, while longer stored samples of refrigerated and room temperature samples trend towards lower PC1 and higher PC2 values. The longest stored room temperature samples appear to be most distant from the samples measured at the beginning of the experiment.

Similarly, the blanched samples are also grouped together (B). However, there is no time-based pattern visible. Longer stored samples do appear further away from samples at day 0 but do not follow a specific direction. Warmer stored samples do move further away with storage time than colder stored samples.

Sliced and intact piece samples are clustered closer together than the other types of samples. A clear area separation between them is not possible. Time-based patterns are not very clear either. There is, however, a rough separation between the storage time dependent vectors of frozen (C) and refrigerated as well as room temperature (D) samples. There are, however, some outliers to this pattern.

Additionally, the plot reveals an estimate of the reproducibility of replicated samples. Generally, they are close together, which indicates good reproducibility. Only occasionally, they are farther apart, most likely due to inconsistencies within the truffle samples.

#### 3.3.2. Interpretation of Trajectories for Identified Compounds

**Figure 5:**
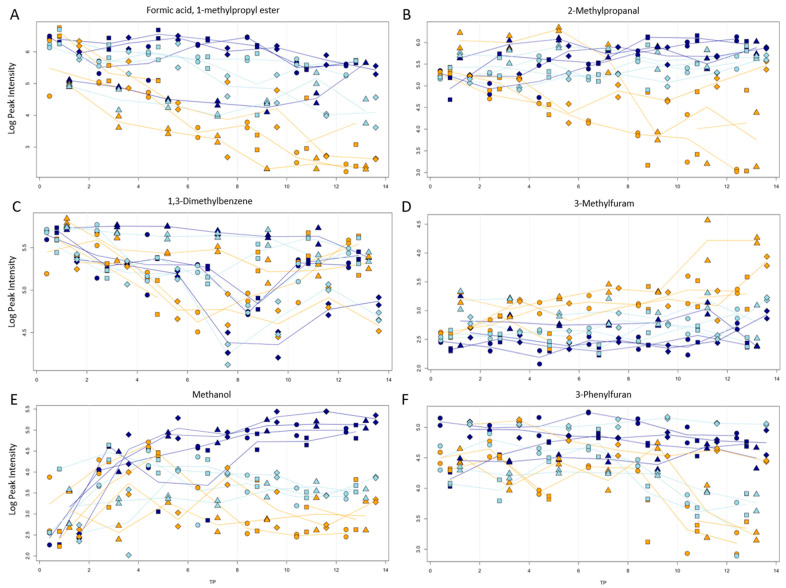
Progression of peak intensity (logarithmic scale) vs. storage time (in days) for formic acid, 1-methylpropyl ester (**A**), 2-methylpropanal (**B**), 1,3-dimethylbenzene (**C**), 3-methylfuran (**D**), Tetrahydrofuran (**E**), and Nonanal (**F**). Storage temperatures of room temperature, fridge, and freezer are indicated by colors. Processing types sliced (●), piece (■), with NaCl (◆), and blanched (▲) are indicated by symbols. To allow easier comparison between groups, lines colored according to temperature levels connect replicate means per time point. Additionally, samples for each time point have been shifted slightly on the *x*-axis according to the applied processing type. Plot annotations provide information on retention time (RT), ion mass (mz), sum formula, peak ID, and the *p*-values obtained in an ANOVA with 3 factors.

Delving deeper into the data, several compounds showed correlations of their peak intensity levels with the variables temperature, time, and processing type. To investigate this further, plots were created that show the progression of the peak intensities with the variables. Plots for all peaks are published online (https://doi.org/10.5281/zenodo.10886445, accessed on 1 March 2024). Figure 5 shows a set of behaviors that were observed in multiple compounds. Note that the *y*-axis is scaled logarithmically and not normalized. Values can only be compared over samples within a metabolite. To this end, a difference of one unit on the *y*-axis represents a relative 10-fold increase. To give an example, in Figure 5B, maximum levels of 6 (equal to 10^6^ intensity) are found for early time points which decrease to levels of 3 (equal to 10^3^ intensity) after 10 days, indicating a 1000-fold decrease in measured levels for this metabolite.

Plot A of formic acid, 1-methylpropyl ester, shows an overall decrease in this compound with storage time. The decrease is also strongest for room temperature stored samples and weakest for frozen samples. Plot B shows a similar behavior for 2-methyl-1-propanal, with the levels for frozen samples being roughly stable. A similar behavior was observed for 2,4-Di-tert-butylphenol, isopropyl formate, 1-(methylthio)-propane, 1-chloro-2-methylbutane, isopropyl formate, propanal, 2-methylbutanal, methanethiol, 2-methylbutyl formate, ethyl formate, and 2-butanol. In some cases, like for 1,3-dimethylbenzene, shown in Plot C, but also p-xylene, ethylbenzene, isobutyl nitrite, 1-propanol and 3-ethylanisole, the processing type played a bigger role than the storage temperature. Here, the decrease was weakest in the blanched samples. A decrease with storage time regardless of storage temperature and condition was observed for 3-octanol, 3-methylphenol, and 2-ethylphenol.

An increase in the aroma compound with time and temperature could also be observed in some cases. Plot D shows this exemplarily for 3-methylfuran. Similar plots were observed for phenyl ethyl ketone, pyrazine, methylpyrazine, 2-dodecanone, 4-heptanone, 1-phenyl-1-pentanone, 2,3-dimethyl-2-cyclopenten-1-one, 2-ethyl-6-methyl-pyrazine, and 2-methylbutanoic acid methyl ester. A more unusual progression can be seen for tetrahydrofuran (plot E). While the amount of tetrahydrofuran increases in the frozen samples, an initial small increase and then a decrease after day 4 were seen for room temperature and fridge stored samples. Nonanal (plot F) showed to be stable until day 8. Only after this time point, a decrease was visible for room temperature and fridge samples, while frozen samples remained stable.

**Figure 6:**
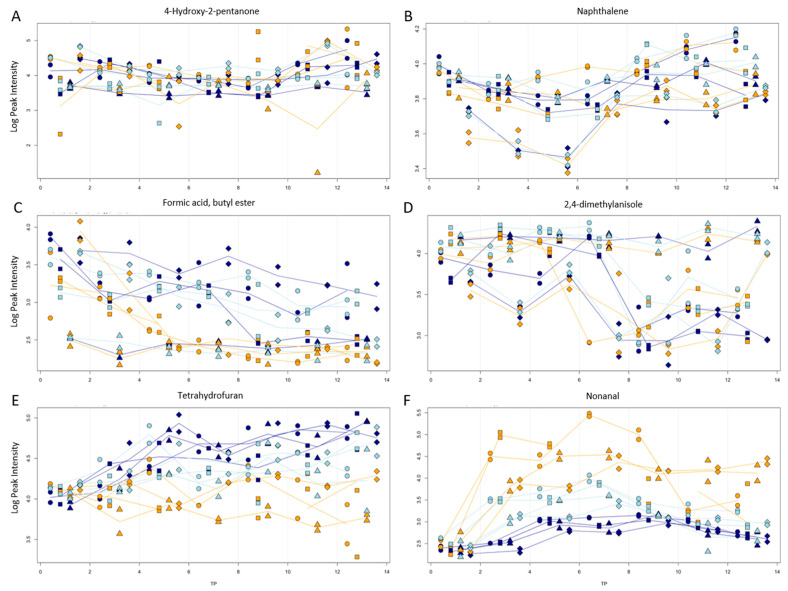
Progression of peak intensity (logarithmic scale) vs. storage time (in days) for 4-hydroxy-2-pentanone (**A**), naphthalene (**B**), formic acid, butyl ester (**C**), 2,4-dimethylanisole (**D**), methanol (**E**), and 3-phenylfuran (**F**). Storage temperatures of room temperature, fridge, and freezer are indicated by colors. Processing types sliced (●), piece (■), with NaCl (◆), and blanched (▲) are indicated by symbols.

Within the numerous compounds that were identified, there were also some that were mostly stable regarding all variables. Figure 6 plot A shows this for 4-hydroxy-2-pentanone. The plots for anisole, methyl isobutyl ketone, and benzaldehyde showed a similar progression. The only compound that showed an initial decrease followed by an increase was naphthalene (plot B).

That the blanching of truffles can have a great impact on their storage ability can be seen in plots C (formic acid butyl ester) and D (2,4-dimethylanisole). In plot C, the blanching ensured the immediate absence of the compound, while in plot D, it ensured the stability of the compounds over time and temperature.

Plot E shows methyl alcohol. It was the only compound that showed an increase in the fridge and freezer samples over time but not at room temperature. 3-phenylfuran in plot F showed stronger effects for increased temperatures and an initial increase, followed by a decrease after day 8.

**Figure 7:**
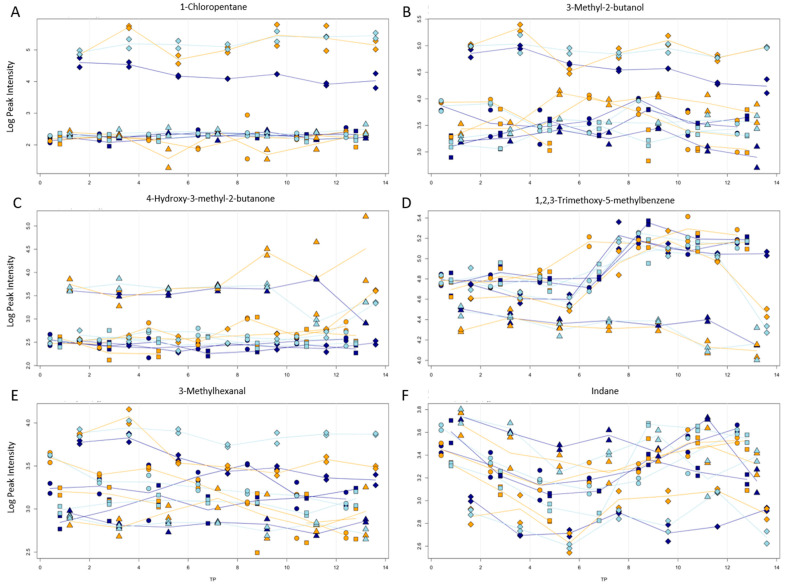
Progression of peak intensity (logarithmic scale) vs. storage time (in days) for 1-chloropentane (**A**), 3-methyl-2-butanol (**B**), 4-hydroxy-3-methyl-2-butanone (**C**), 1,2,3-trimethoxy-5-methylbenzene (**D**), 3-methylhexanal (**E**), and indane (**F**). Storage temperatures of room temperature, fridge, and freezer are indicated by colors. Processing types sliced (●), piece (■), with NaCl (◆), and blanched (▲) are indicated by symbols.

The compounds which variance correlated strongest with the processing types are depicted in Figure 7. There were some that were only present in the NaCl-treated samples like 1-chloropentane, (plot A), or are much higher in NaCl-treated samples like 3-methyl-2-butanol, (plot B). The effect was less prevalent in frozen samples. Other compounds that showed similar plots were 3-methylformamide, tributyl phosphate, 1-chloroheptane, isobutyl valerate, 1-chlorobutane, 2-methylbutyl hexanoate, isobutyl hexanoate, propyl hexanoate, 2-chloro-3-methylbutane, 4-ethyl-3-nonen-5-yne, 2-methylbutanoic acid, 3-Octen-2-one-pentyl ester, (E)-2-heptenal, 2-methylpentyl isobutyrate, pentanal, 2-ethyl-2-hexenal, 2-ethyl- 4-pentenal, and hexanoic acid.

Fewer compounds showed a correlation with blanching. 4-hydroxy-3-methyl-2-butanone (plot C) and 2-methyl-2-Pentanol were the only ones that were present in blanched samples but not or almost not in all other samples. On the other hand, the blanching led to a significantly lower concentration for 1,2,3-trimethoxy-5-methylbenzene (plot D), 1,2,3-trimethoxybenzene, Hexyl 2-butenoate, Benzene, 1,2,3-trimethoxy-5-methyl-, and 3,4-Dimethoxytoluene.

A correlation with both NaCl treatment and blanching was present for Hexanal, 3-methyl- (plot E), 1-Pentanol, Acetylacetone, formic acid, hexyl ester and 1-Hexanol. For these, the concentration was higher in NaCl samples and lower in blanched samples. A higher amount for both treatments was observed for 2,3-Butanedione, 3-Penten-2-one, 3-methyl- and 2-Butenal, 2-ethyl-. The only compound with a lower concentration for NaCl samples was indane (plot F).

Volatile sulfur compounds (VSCs) are a class of aroma substances that are more specific to truffles. In the literature, these are described to be important for the overall aroma perception in humans and animals and are related to biological processes during their maturation. Therefore, special attention was paid to them in this study.

**Figure 8:**
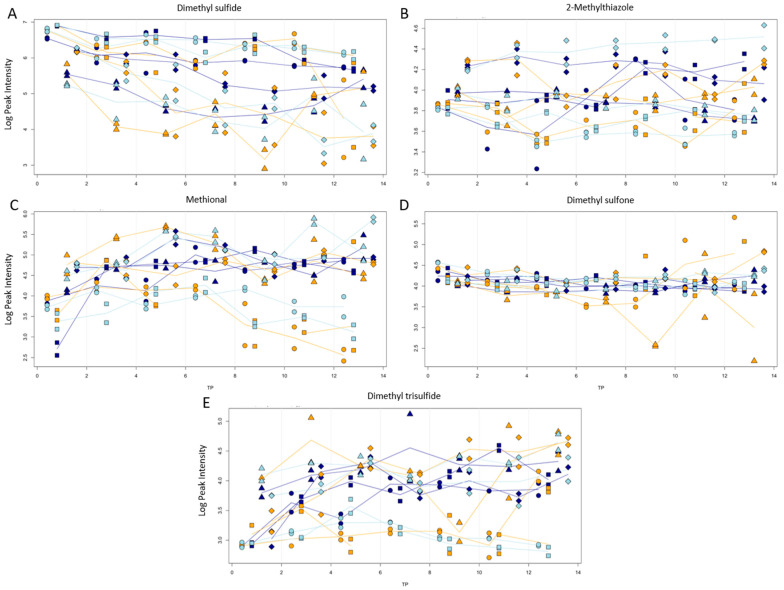
Progression of peak intensity (logarithmic scale) vs. storage time (in days) for dimethyl sulfide (**A**), 2-methylthiazole (**B**), methional (**C**), dimethyl sulfone (**D**), and dimethyl trisulfide (**E**). Storage temperatures of room temperature, fridge, and freezer are indicated by colors. Processing types sliced (●), piece (■), with NaCl (◆), and blanched (▲) are indicated by symbols.

Dimethyl sulfide, one of the most frequently found VSCs in truffles, shows an overall decrease in its intensity over time (Figure 8A). A similar trend was noticeable for dimethyl sulfoxide, methanthiol, 1-(methylthio)-propane, 2-(methylthio)-ethanol, and 2,4-dithiapenthane.

Slightly elevated levels in NaCl samples, but a mostly stable progression in regard to the storage time, was found for 2-methyl-thiazole (plot B) and 2-methyl-thiophene. A decrease after day 8 for room temperature and fridge samples was observable for methional (plot C). The oxidized form of dimethyl sulfide, dimethyl sulfone (plot D), showed mostly stable levels with only some variance at longer storage durations. A significantly lower intensity for sliced and pieced room temperature and fridge samples over the entire storage time was visible for dimethyl trisulfide.

### 3.4. Enrichment and Pathway Analysis

Although these results already simplified the vast amount of data collected, there are still pieces of information in these kinds of datasets that are hard to manually analyze. Of particular interest in this case is the correlation of compound data with their biological functions. For this purpose, the software MetaboAnalyst 6.0 was used to investigate the enrichment of relevant metabolites in chemical classes and metabolic pathways. Because we are interested in time-dependent metabolic changes, relevant metabolites were defined as showing a statistically significant difference according to time, internally calculated as an ANOVA *p*-value by MetaboAnalyst (cf. Methods) [52,53].

**Figure 9:**
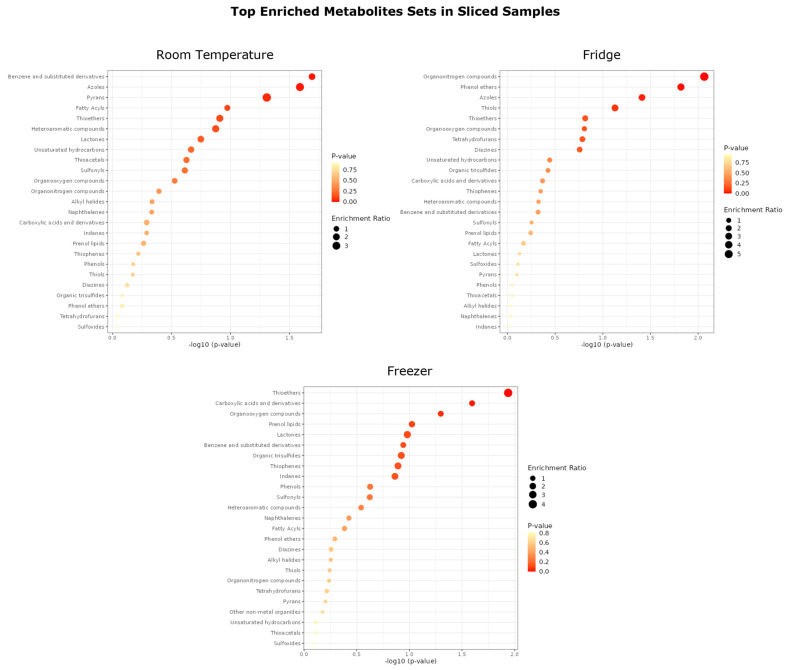
Top enriched metabolites by main chemical class in sliced samples, divided by storage temperature.

Exemplary, the enrichment analysis of sliced samples divided by storage temperatures is shown in Figure 9. The enrichment ratios are shown as circle sizes, and the *p*-values are color-graded.

The aroma of truffles may be produced by truffles themselves or by the microbial community that colonizes them, made up of bacteria, yeasts, guest filamentous fungi, and viruses [14,54]. Since a metabolite library for either truffles (*tuber*) or a truffle-specific microbial community was not available, the enrichment set was classified by main chemical classes for this analysis.

From Figure 9, the enrichment of compounds varies with the temperature. Organosulfur compounds like thioethers, thiophenes, or sulfonyls are enriched to a greater extend with decreasing temperature. The same is true for organooxygen compounds. At room temperature, benzene and substituted derivatives, fatty acyls, and heteroaromatic compounds are more enriched than at lower temperatures. Noticeable is that N-containing compounds like azoles seem to be enriched at all storage conditions. Phenols, phenol ethers, and tetrahydrofurans are among the least-enriched compounds overall.

**Figure 10:**
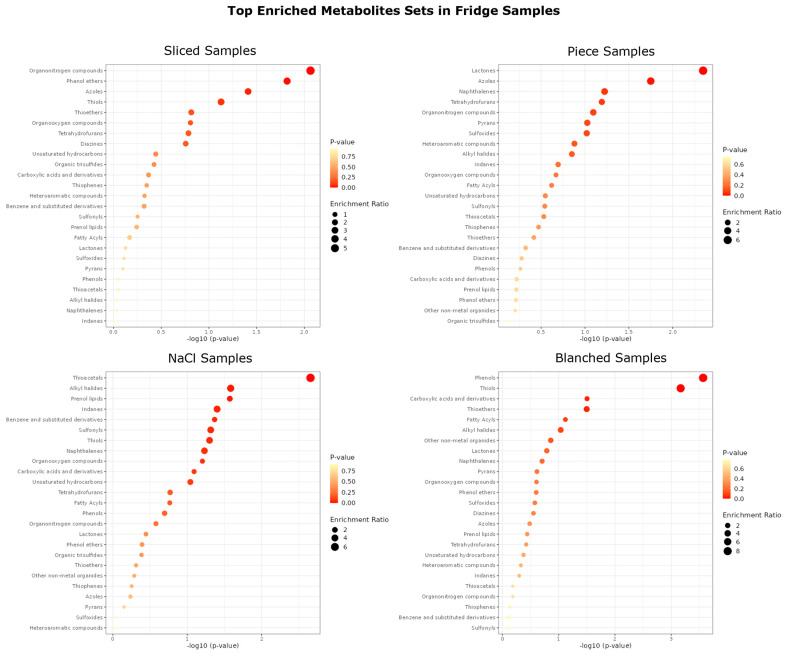
Top enriched metabolites by main chemical class in fridge samples, divided by treatment method.

In Figure 10, the top enriched metabolites in fridge stored samples are shown.

Proceeding from piece samples, all other treatments seem to result in a greater enrichment of some sulfur-containing compounds. For sliced samples, these compounds are thiols and thioethers; for NaCl samples, they are thioacetals sulfonyls and thiols; and for blanched samples, the most-enriched s-compounds are thiols and thioethers. Compared to that, the most-enriched S-compounds in piece samples are sulfoxide. There are also differences among the other chemical structure classes between the sample types, but a clear pattern is not emerging.

**Figure 11:**
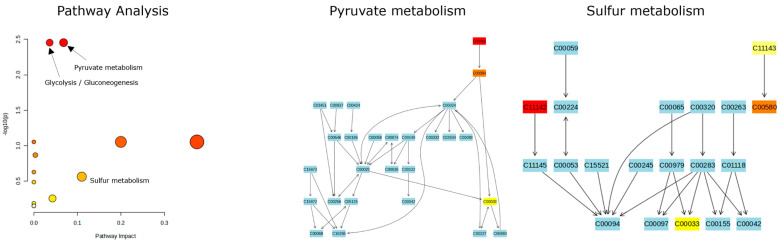
Pathway analysis of room temperature sliced samples and selected metabolite pathways that include more than 2 identified compounds.

To gain insight into what the enrichment of compounds means for the biological processes occurring in the stored truffe samples, a pathway analysis was run. In Figure 11, the results for sliced samples stored in the fridge are shown as an example. The analysis was run against the metabolomic pathway library of Escherichia coli K-12 MG1655 as a model bacterium. This was performed since no library for truffles or truffles specific bacteria was available.

On the left, the logarithmic *p*-value vs. the pathway impact plot is shown. The highest *p*-value observed was for the pyruvate and/or glycolysis pathway. The pyruvate pathway is also depicted in Figure 10. The compounds that participate in both pathways are ethanol (C00469), acetaldehyde (C00084), and acetate (C00033). Although there are some enriched compounds that are part of other pathways, only two or less of the identified compounds take part in these. Therefore, the pathway impact shown here is not very reliable. This is to be expected, since these data stem from GC–MS measurements, and the libraries were mostly carried out with/for LC–MS analyses. The only other pathway that was impacted by multiple enriched compounds is the sulfur metabolism. Dimethyl sulfone (C1142), acetate (C1143), dimethyl sulfoxide (C00580), and dimethyl sulfone (C00033) take part in this metabolism.

## 4. Discussion and Conclusions

The presented data showcase the influence of different factors on the aroma of truffles. While pre-storage processing methods that target the enzymatic activity or heat treatments can alter the aroma of truffles fundamentally, the surface area of truffle samples also plays some role in the storability. The approach of this work was to examine the influence of treatment methods, storage times, storage temperatures, and their interactive effects on individual aroma compounds that make up the aroma of *Tuber melanosporum*.

Tracking the changes in the aroma, 695 compounds could be identified which concentrations varied throughout the experiment. These comprise far more compounds than other studies, which mainly focusing on the aroma profiling at a given point in time had previously found. This emphasizes the complexity of processes happening in truffles regarding different factors.

The results can be used to further develop the understanding of truffles and for different applications. Principle component analyses of aroma profiles may give a first and simple insight into the truffle constitution. Individual compounds in the truffle aroma could be utilized to comprehend the treatment samples underwent in the past.

The content of formic acid, 1-methylpropyl ester, may be used to determine the overall age of *Tuber melanosporum* samples as this compound’s concentration decreases with time. 2-Methyl-1-propanal, 1-(methylthio)-propane, or 2,4-dimethylanisole may be considered more as freshness markers as their decrease is weakened with a decrease in storage temperature. Increased levels of tetrahydrofuran can indicate that a truffle was frozen at one point. The presence of 1-chloropentane can be used to reconstruct the contact or treatment with salt, while 4-hydroxy-3-methyl-2-butanone may indicate a previous heat treatment.

Also, the information about time- and temperature-stable concentrations of, for example, 4-hydroxy-2-pentanone and dimethyl trisulfide may be utilized in future works to correlate with similar information about other truffle species.

Compounds found often in truffle literature like 3-methylbutanal, 2-butanone, 1-octen-3-ol, 2-butenal, acetaldehyde, 3-methyl-1-butanol, and 3-octanol could also be identified in this study but showed no interesting progression in their concentration. Others like 2-methylbutanal, 3-methyl-1-butanal, anisole, or 6-methyl-2-heptanol were not found here.

In comparison to other studies mentioned in the Introduction, a strong alteration of truffles after freezing, but comparably well preservation by heat treatment, could not be confirmed in this study. This study tends to agree with those who found any heat treatment to have a strong effect on the aroma of truffles. On the other hand, markers for freshness such as dimethyl sulfide and others could be confirmed. It was further found that a treatment with NaCl had the strongest effect on the aroma profile of *Tuber melanosporum*.

Since the focus of this work, as well as the authors’ knowledge and abilities, are limited, we provide all collected data for others to use (https://doi.org/10.5281/zenodo.10886445, accessed on 1 March 2024). This could for example open the possibility of a more in-depth interpretation of the collected data based on biological processes. As an example, we provided a metabolite enrichment and pathway analysis of some of the samples. This is a way to identify biologically meaningful patterns that are significantly enriched. This approach directly investigates if a set of functionally related metabolites are significantly enriched without the need to manually preselect compounds based on an arbitrary cut-off threshold. It has the potential to identify subtle but consistent changes among a group of related compounds. From the results, it is again clearer that sulfur compounds play an important role in the truffle aroma. Significant differences in their enrichment at different storage temperatures and different sample treatments could be observed.

The impact of some of the enriched compounds on metabolic pathways could be observed. Although the analysis was limited by the accessible metabolomic libraries, others with a better understanding and knowledge in this field could potentially gain much more information on biological processes from data collected in this study.

Overall, the results show how different factors like temperature and sample treatment methods impact the aroma of *Tuber melanosporum* over an extended storage time. Markers for these factors could be identified. The correlation of the collected data with biological processes showed to be promising for the future.

## Data Availability

The original data presented in the study are openly available in Zenodo at https://doi.org/10.5281/zenodo.10886445, accessed on 5 March 2024.

## Appendix A

**Table A1:** List of all samples prepared for storage.

Sample Nummer	Storage Temperature	Processing Type	Storage Time	Repetition
1	TK	S	0	1
2	TK	S	0	2
3	TK	B	0	1
4	TK	B	0	2
5	TK	SA	0	1
6	TK	SA	0	2
7	TK	BL	0	1
8	TK	BL	0	2
9	RT	S	0	1
10	RT	S	0	2
11	RT	B	0	1
12	RT	B	0	2
13	RT	SA	0	1
14	RT	SA	0	2
15	RT	BL	0	1
16	RT	BL	0	2
17	KS	S	0	1
18	KS	S	0	2
19	KS	B	0	1
20	KS	B	0	2
21	KS	SA	0	1
22	KS	SA	0	2
23	KS	BL	0	1
24	KS	BL	0	2
25	TK	S	2	1
26	TK	S	2	2
27	TK	B	2	1
28	TK	B	2	2
29	TK	SA	2	1
30	TK	SA	2	2
31	TK	BL	2	1
32	TK	BL	2	2
33	RT	S	2	1
34	RT	S	2	2
35	RT	B	2	1
36	RT	B	2	2
37	RT	SA	2	1
38	RT	SA	2	2
39	RT	BL	2	1
40	RT	BL	2	2
41	KS	S	2	1
42	KS	S	2	2
43	KS	B	2	1
44	KS	B	2	2
45	KS	SA	2	1
46	KS	SA	2	2
47	KS	BL	2	1
48	KS	BL	2	2
49	TK	S	4	1
50	TK	S	4	2
51	TK	B	4	1
52	TK	B	4	2
53	TK	SA	4	1
54	TK	SA	4	2
55	TK	BL	4	1
56	TK	BL	4	2
57	RT	S	4	1
58	RT	S	4	2
59	RT	B	4	1
60	RT	B	4	2
61	RT	SA	4	1
62	RT	SA	4	2
63	RT	BL	4	1
64	RT	BL	4	2
65	KS	S	4	1
66	KS	S	4	2
67	KS	B	4	1
68	KS	B	4	2
69	KS	SA	4	1
70	KS	SA	4	2
71	KS	BL	4	1
72	KS	BL	4	2
73	TK	S	6	1
74	TK	S	6	2
75	TK	B	6	1
76	TK	B	6	2
77	TK	SA	6	1
78	TK	SA	6	2
79	TK	BL	6	1
80	TK	BL	6	2
81	RT	S	6	1
82	RT	S	6	2
83	RT	B	6	1
84	RT	B	6	2
85	RT	SA	6	1
86	RT	SA	6	2
87	RT	BL	6	1
88	RT	BL	6	2
89	KS	S	6	1
90	KS	S	6	2
91	KS	B	6	1
92	KS	B	6	2
93	KS	SA	6	1
94	KS	SA	6	2
95	KS	BL	6	1
96	KS	BL	6	2
97	TK	S	8	1
98	TK	S	8	2
99	TK	B	8	1
100	TK	B	8	2
101	TK	SA	8	1
102	TK	SA	8	2
103	TK	BL	8	1
104	TK	BL	8	2
105	RT	S	8	1
106	RT	S	8	2
107	RT	B	8	1
108	RT	B	8	2
109	RT	SA	8	1
110	RT	SA	8	2
111	RT	BL	8	1
112	RT	BL	8	2
113	KS	S	8	1
114	KS	S	8	2
115	KS	B	8	1
116	KS	B	8	2
117	KS	SA	8	1
118	KS	SA	8	2
119	KS	BL	8	1
120	KS	BL	8	2
121	TK	S	10	1
122	TK	S	10	2
123	TK	B	10	1
124	TK	B	10	2
125	TK	SA	10	1
126	TK	SA	10	2
127	TK	BL	10	1
128	TK	BL	10	2
129	RT	S	10	1
130	RT	S	10	2
131	RT	B	10	1
132	RT	B	10	2
133	RT	SA	10	1
134	RT	SA	10	2
135	RT	BL	10	1
136	RT	BL	10	2
137	KS	S	10	1
138	KS	S	10	2
139	KS	B	10	1
140	KS	B	10	2
141	KS	SA	10	1
142	KS	SA	10	2
143	KS	BL	10	1
144	KS	BL	10	2
145	TK	S	12	1
146	TK	S	12	2
147	TK	B	12	1
148	TK	B	12	2
149	TK	SA	12	1
150	TK	SA	12	2
151	TK	BL	12	1
152	TK	BL	12	2
153	RT	S	12	1
154	RT	S	12	2
155	RT	B	12	1
156	RT	B	12	2
157	RT	SA	12	1
158	RT	SA	12	2
159	RT	BL	12	1
160	RT	BL	12	2
161	KS	S	12	1
162	KS	S	12	2
163	KS	B	12	1
164	KS	B	12	2
165	KS	SA	12	1
166	KS	SA	12	2
167	KS	BL	12	1
168	KS	BL	12	2
